# Towards large-scale analyses of settlement patterns in urbanizing landscapes—findings of first studies for India, Egypt, and China

**DOI:** 10.1038/s41598-024-74678-w

**Published:** 2024-10-28

**Authors:** Thanh Thi Nguyen, Thomas Esch, Ellen Hoffmann, Julian Zeidler, Lorenz Gruber, Dennis Kaiser, Andreas Buerkert

**Affiliations:** 1 Organic Plant Production and Agroecosystems Research in the Tropics and Subtropics, Faculty of Organic Agricultural Sciences, University of Kassel, 37213 Witzenhausen, Germany; 2 German Aerospace Center (DLR), German Remote Sensing Data Center, Land Surface Dynamics, Oberpfaffenhofen, 82234 Weßling, Germany; 3 Department of Computer Science, University of Wuerzburg, 97074 Wuerzburg, Germany; 4 Faculty of Geomatics, Computer Science and Mathematics Photogrammetry and Geoinformatics, University of Applied Sciences (HFT), Schellingstr. 24, 70174 Stuttgart, Germany

**Keywords:** Rural-urban transformation, Settlement pattern, Graph, Fractal analysis, Automation, Information technology, Environmental sciences, Environmental social sciences, Planetary science

## Abstract

Improved ability to assess and categorize the spatial characteristics of settlement patterns is required for a deeper understanding of how urbanization is driving land use and land cover transformation and its effects. Two approaches to the globally available settlement maps of the World Settlement Footprint 3D support a detailed assessment of spatial characteristics of settlement patterns in rural to urban landscapes and across scales: graph-based spatial network analysis and elements of fractal theory. Based on first comprehensive tests for the Punjab (India), the Nile Delta (Egypt) and the North China Plain, the results of our study suggest that the presented methods allow a quantitative and qualitative characterization and comparison of settlement patterns between different regions of the world. The approache allows to generate standardized baseline data for arbitrary regions in the world to analyze structuring principles of settlement hierarchies (e.g., self-organized fractal geometries) and their dependence on - or interaction with - cultural, political, socioeconomic, or environmental conditions.

**Supplementary Information:**

The online version contains supplementary material available at 10.1038/s41598-024-74678-w.

## Introduction

The last decade has seen rapidly growing interest in urbanization and rural-urban transformation processes, with numerous approaches being employed globally^1–3^. Remote sensing has become a widely used tool to trace settlement growth patterns and dynamics, with most analyses focusing on cities and their immediate surroundings^4,5^, or on rural-urban gradients/continua^6–8^ whereby rural regions are rarely considered^9,10^. The concept of “*rurbanity*”^11^ calls for abandoning the dichotomy of rural and urban areas. It requires a methods of settlement pattern analysis that transcend urban to rural landscapes and spatial scales, especially in countries of the Global South. There urbanization proceeds more rapidly than elsewhere, but secondary (demographic and statistical) data are often scarce^2,11^. In such context, the analysis of land use patterns – here, in particular the extend of human settlements - holds significant promise in providing clues to understanding key transformation processes and their drivers^2,12^.

Among the previous, mainly data-driven approaches to characterize large scale settlement patterns, graph-theory and spatial network analysis based on settlement maps such as the World Settlement Footprint^13,14^ proved to have high potential^15–17^. This method allows to capture and analyze the full range of settlement sizes from megacities to small villages, along with their spatial relations. It effectively visualizes urban agglomerations, corridors connecting them, transitional areas and remote rural regions. It describes them by quantitative, network-derived indices, that express relative importance and hierarchical relations. A different, rather theory-driven approach which aimed to detect principles of self-similarity with fractal features across scales in an exemplary settlement pattern in a large scale irrigation scheme was introduced by Nguyen et al.^12^ Fractal patterns are formed, mathematically, by iteration of an initial shape, divided by a fixed factor in each iteration step, which results in self-similarity at different scales of magnification or resolution^18^. They are widespread in nature, with snow flakes or fern leaves as frequently cited examples^19^, and are thought to emerge from self-organizing physical or biological processes. Fractal principles have also been applied purposefully for city planning to create ‘organic’ structures that provide more pleasant conditions for the inhabitants^20^. The Fractalopolis model, used in our study to assess overall spatial landscape configurations, was originally developed for such a purpose^21^, while Nguyen et al.^12^ applied it as an analytical/diagnostic tool.

The main objective of the present study is to jointly apply the analysis of graph-based spatial networks and of fractal features to three different study regions which show apparently similar spatial patterns^12^: the Indian Punjab, the Nile Delta in Egypt, and the North China Plain. With such a large-scale combined analysis we expect to enhance the understanding of settlement patterns and how they emerge from formation and transformation processes driven by urbanization^22^. At the same time, we want to increase the degree of automation of the underlying workflows as a basis for future implementation of a fully operational framework that integrates data-driven and theory-driven elements and can be applied to arbitrary areas of interest around the world at different levels of scale.

## Methodology

### Data

As baseline data to conduct the settlement hierarchy and pattern analyses, we use the World Settlement Footprint 3D (WSF 3D) dataset introduced by Esch et al.^14^ and available for download at https://download.geoservice.dlr.de/WSF3D/files/.

### Network generation

The workflow to construct and analyze the spatial settlement network is based on the methodology presented by Esch et al.^15^. However, the performance of the network generation is now comprehensively improved by applying a sweep line approach to retrieve nearby settlements. This method comprises a algorithmic complexity and makes the proposed method applicable for big data processing. First, settlement polygons are generated by means of image segmentation based on the WSF 3D raster data. Next, all polygons with an area < 20,000 m^2^ are removed to avoid that single houses and isolated homesteads are counted when determining the number of settlements, and their total and average area in the regions of interest. The Jenks Natural Breaks data clustering^23^ is then applied to subdivide the distribution of all settlement areas within each study region into five size classes (XL, L, M, S, XS; see the Results section). This introduces a stratification by size into the network generation in order to detect fractal-like hierarchical features. Subsequently, nine networks are generated per study region – one network each for the five settlement size classes (XL, L, M, S, XS) and four additional networks with objects of one class plus the next lower one (XL-L, L-M, M-S, and S-XS). They form the basis for the subsequent settlement pattern analysis. Next, a buffer has to be defined for connecting the segmented settlement patches. Generally, the buffer distance should be chosen such that it connects all relevant neighbors while at the same time avoiding that too many objects far outside the primary neighborhood are linked (this avoids that the number of arcs - and therefore the complexity of the network - gets too high). For the present type of analysis, an approximate determination of the initial buffer distance is sufficient, since the network indices later used for the pattern analysis are either not sensitive to small deviations, or the network created can be thinned out again if necessary. We set the buffer distance to 200 km for connecting the XL settlements, 100 km for L, 20 km for M, 10 km for S and 5 km for XS. Here it is important to note that the use of fixed buffer sizes is not fully generalizable and the chosen settings may therefore not be suitable for every region of the world. A first proposal for improving this processing step is given in Sect. 4.4 and will be implemented as part of a follow-up study.

### Network analysis

Esch et al.^15^ provided a comprehensive collection of parameters to describe the properties of the settlement objects representing the nodes of the network (area, perimeter, shape index, solidity), the arcs/edges connecting these nodes (length, direction) and local network-related indices calculated from the characteristics of the nodes and their related arcs (such as degree centrality, local significance, local nearness). While degree centrality is solely based on the number of edges connected to a settlement, measures like local significance take spatial features into account. The local significance of an area between two settlements is defined as the ratio of the multiplied areas to the distance between the nodes. We applied this approach to our study areas and used selected parameters for a comparative analysis of the study regions (Table 1). Measures within the same size stratum and between subsequent strata were taken in order to derive parameters for fractal projections.

### Projection of fractals

The construction of fractal projections using the Fractalopolis model^21,24^ requires input parameters such as the number of size classes, sub-centers, distances and median angles, all of which can in principle be derived from the network analysis. However, the values determined here through the automated processes were found to be unsuitable (see Results section). We instead derived the parameters from one randomly selected site in each landscape (Nile Delta and North China) and iteratively applied the Sierpinski carpet rule^18,25^ to visualize the corresponding fractal. By shifting this pattern to other equivalent settlement centers, we (visually) assessed the fit of the entire landscape to the theorized self-similarity pattern^12^.

## Results and discussion

The study regions selected in the North China Plain and the Nile Delta are similar in size, while that in the Punjab is much larger (see Table 1). All three regions are densely inhabited, with 7% of the total area being built-up in the Punjab, 9% in the Nile Delta, and 21% in China. All of them show qualitatively similar settlement patterns of differently sized cities with apparently regular spacings between them that are reminiscent of a Sierpinski carpet^12^. The total number of detected settlements was highest in the Punjab, but its settlement density was less than half of that in the other two regions (3.6 per 10 km^2^ as compared to 8.2 in the Nile delta and 7.8 in China). Across all areas the vast majority of settlements falls into the lower size classes. S and XS settlements account for 88% and 99% of all settlements in the Punjab and Nile Delta, respectively, and for 57% in the North China Plain (Table 1). These results already indicate quantitative differences in the pattern parameters.

**Fig. 1:**
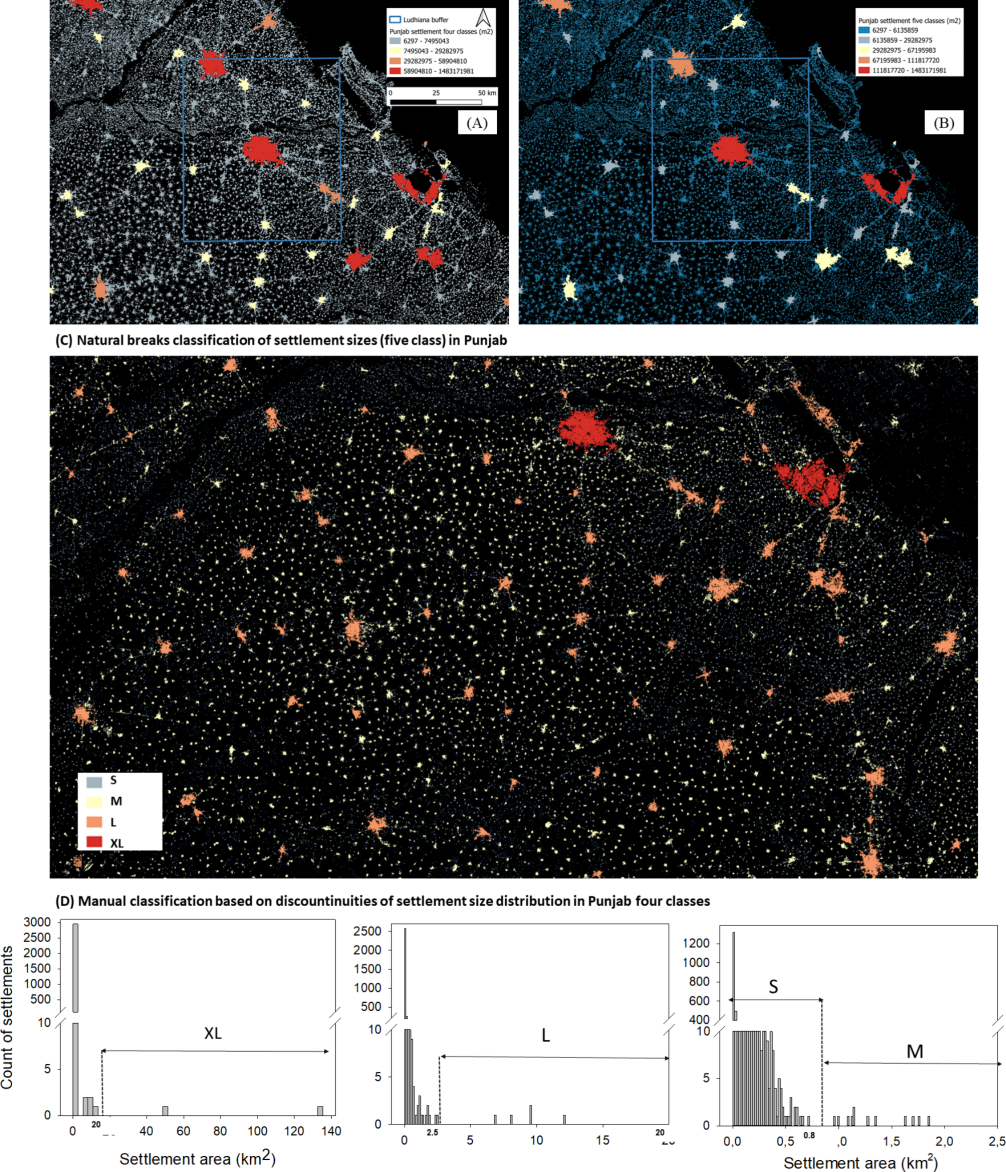
Differences of automatic classification with four classes (**A**) and five classes (**B**). Four (out of five) settlement classes using (**C**) natural breaks with automatic classification of settlement classes *versus* (**D**) manual classification in the Punjab (Nguyen et al.^12^). Maps were generated using QGIS 3.22.

*Settlement hierarchy* - The results of the automatic natural breaks categorization of settlements into five classes yielded different break points in the three regions (Fig. 1; Table 1, Annex). The size of the largest cities (XL) differed considerably: The Nile Delta was dominated by the megacity of Cairo with ca. 1.200 km^2^, whereas the XL class comprised eight cities with an average size of ca. 350 km^2^ in the Punjab, and 56 cities of ca. 50 km^2^ in the North China Plain.

The average distance to the next three neighboring settlements of the same size class decreased steadily from XL to S class (M in the China case), but then increased again towards the XS class. The increasing distance contradicts the rules of a fractal pattern, but may be an artefact of the size classification, that is S and XS (M to XS in the China case) might actually belong to the same size class. The average size of the smaller settlements was also quite similar across all regions, but especially in the North China, where the M-, S-, and XS-classes were all below one km^2^. The initial size classification is critical for all subsequent steps (Fig. 1), and fixing the natural breaks to five classes thus introduced a bias to the subsequent fractal analysis.

**Fig. 2:**
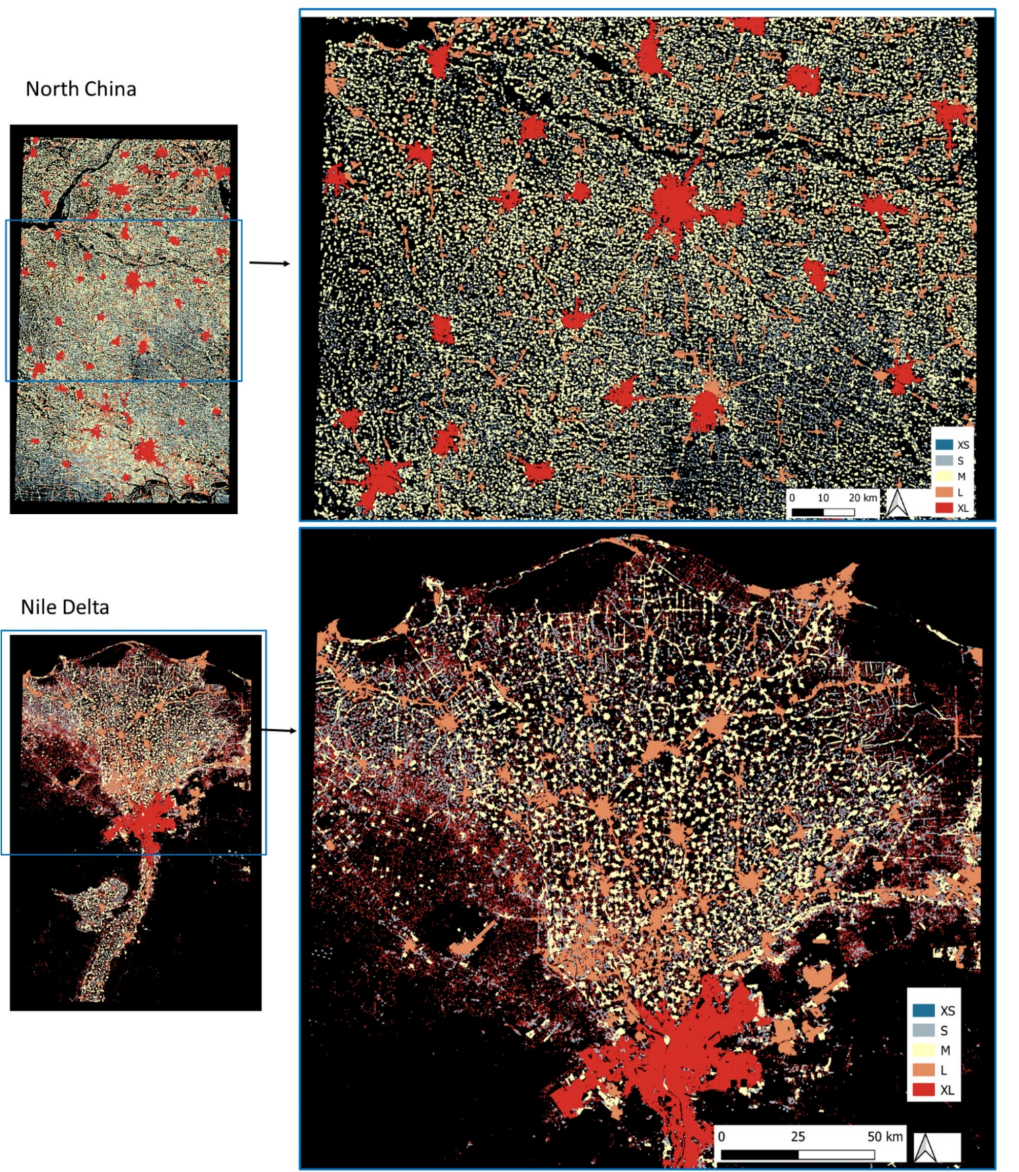
Fixed five settlement size classes in the North China and the Nile Delta using Jenks Natural Breaks^23^. All maps were generated using QGIS 3.22.

Based on these contradictory results, it may be possible to re-group the classes manually, and merge, for example, S and XS in the Punjab and Nile Delta case to four classes in total, and M to XS in the China example to three classes only – or to re-run the automatic classification with the lower number of fixed classes. Either way represents a combination of approaches, where in particular the labor intense steps of defining buffer zones and detecting and classifying settlements are automated (Fig. 2).

The number of neighbors, as well as the number of connections generated in the network, is sensitive to the size of the search buffer. The search buffers defined here were based on the experience in the network analysis^15^. The procedure was useful to determine the average distances between same class neighbors, but turned out to be too large for extracting fractal relationships as it detected too many neighbors of the next lower class (Fig. 1, Annex).

**Fig. 3:**
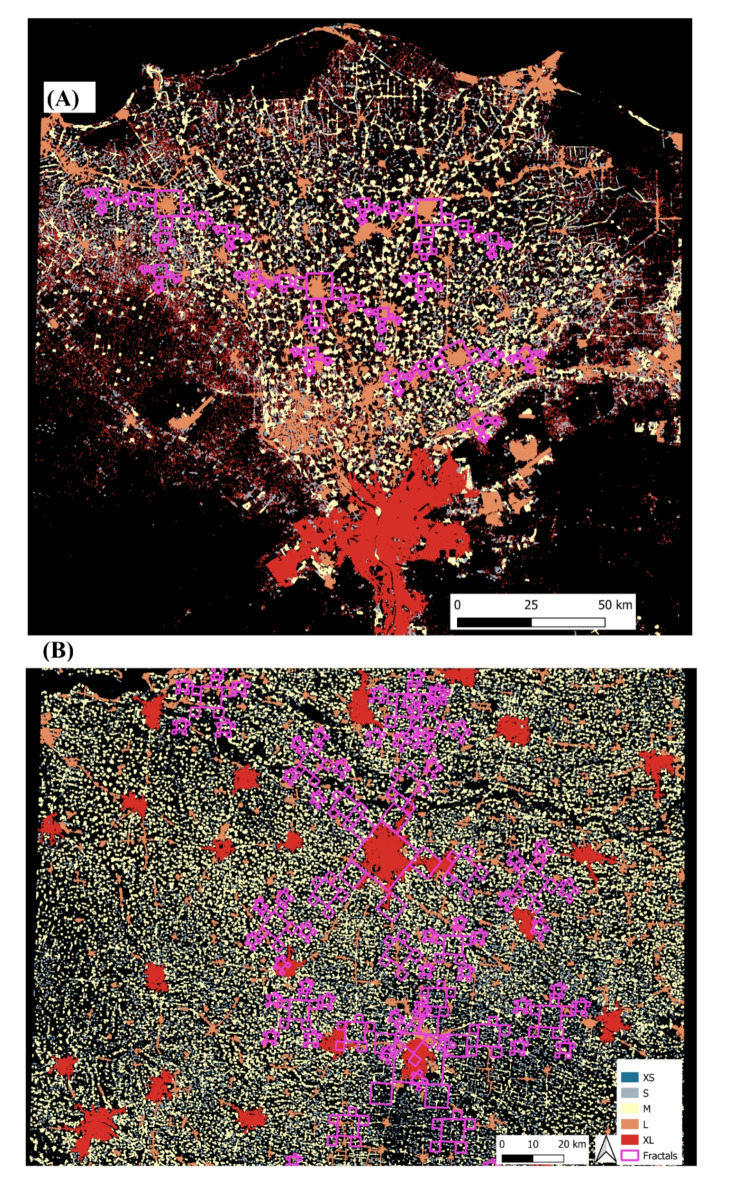
Projected fractals in the Nile Delta (**A**) and the North China Plain (**B**) using Fractalopolis 1.1. All maps were generated using QGIS 3.22, fractals were exported from Fractalopolis then overlaid on maps.

With the same buffer size being used in all regions, the the numbers of connections were also sensitive to the size of the study area. More connectionss were found in the area of North China than Punjab, the larger study areas. These parameters expressed the overall density of the settlement pattern, but they may vary with the geographical conditions; the entire Punjab study site consists of some areas with less density in the Eastern and Western parts (Fig. 1) while in North China (Fig. 3B) the entire region has a quite homogeneous settlement distribution. Herein, the order of area size is North China < Punjab while the order of total number of connections is Punjab < North China.

Following the approach of Esch et al.^15^, we further measured parameters such as the completeness of connections between all two contiguous classes, degree centrality, local significance, local nearness (not shown). They may provide alternatives or amendments to simple settlement size in order to improve the hierarchical classifications. In this study, we focused on the variables which allow us to compare and link the approaches of Esch et al.^15^ and Nguyen et al.^12^.

**Table 1:**
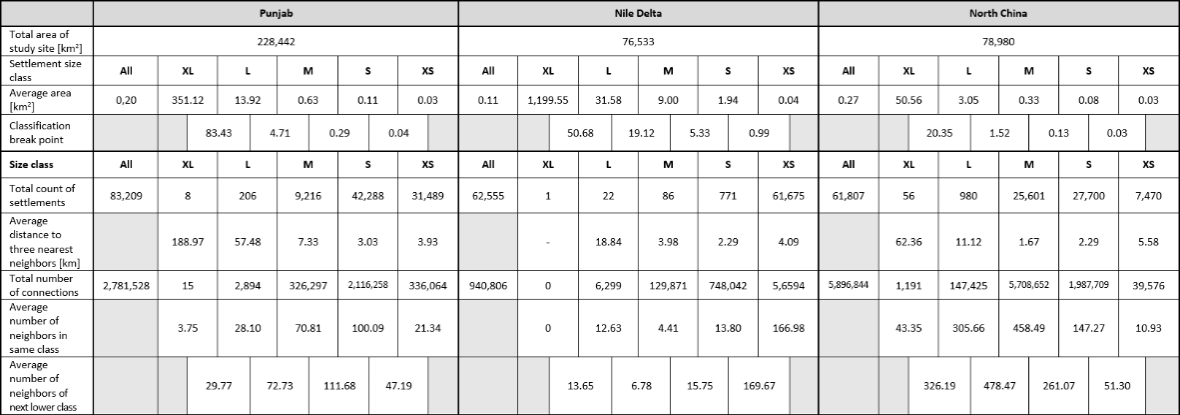
Selected parameters for characterizing the settlement patterns in the three study regions of India, Egypt, and China.

*Projection of fractals* – Fractal patterns show self-similarity across scales, and can be constructed from a few parameters (distances and angles between nodes in a hierarchical network) by iteration, as for example in the Fractalopolis model^21,24^. If such ideal patterns are projected on a real landscape, their congruence can be visually assessed. The pre-defined buffer zones used in the automated spatial measurements detected a high number of sub-centers: 15–20 as compared to 4–5 sub-centers between XL-L and L-M in the earlier study of Nguyen et al.^12^. This confounded the calculation of average distances and angles. We therefore extracted the parameters required for the Fractalopolis model from a single random site in each landscape, starting from the highest settlement size class and its successive lower-class neighbors. This pattern was then replicated and projected around other large settlements in the same landscape. For the Nile Delta, three out of 12 sub-centers were captured by the fractals projected from five centers. Others were close to the projected sites, but would have required a slight shift in the radial axes. The projection of North China’s fractals showed a better fit with approximately 50% of the 15 sub-centers correctly projected at the XL-L class size. Overall, the projections underscore a fractal-like regularity in the spacing and orientation, especially for the first and second order size classes. As for the findings by Nguyen et al.^12^, the settlements within the small classes deviate from the fractal hierarchy and are rather evenly distributed (Fig. 2).

As the aggregated area of small settlements (less than two km^2^ comprised in M to XS classes) still contributes 30–60% of the total built-up area in the regions, we suggest that more attention is needed for micro-urbanization in the Global South and in data-scarce environments^26^. At the global scale, linkage of landscape patterns and rural-urban indices should be explored^6,16^.

Our findings demonstrate the potential of upscaling network analyses combine with the use of fractal theory to understanding urban landscapes. Urban planners should be encouraged to consider natural fractal organization patterns in urban growth processes across different scales. Our findings (Table 1; Fig. 3) support a better understanding of urban landscapes, which may emerge from the combination of complex factors such as social, political, cultural, economic, natural, and other undefined factors, all of which change over time^24,27^.

## Conclusion and outlook

The increased automation of settlement network generation achieved in this study by improving the coding and optimizing the coupling of workflows introduced by Esch et al.^15^ advances the effectiveness and efficiency of pattern analysis. Our new version is now capable of handling and processing terabytes of data, generating large-scale and complex spatial networks (consisting of millions of objects and connections) by leveraging a deployment at High-Performance Clusters (HPC), calculating various graph-based metrics such as areas of and distances between settlements, and network-related indices such as degree centrality, local significance, or local nearness. The large-scale utilization of these parameters already allows a more comprehensive and in-depth analysis of settlement patterns.

Nevertheless, (as pointed out in Esch et al.^15^) it is necessary to link such results to concepts and theories of urban planning or hierarchies of settlements, in order to gain a deeper understanding of the underlying processes of urbanization. The detection of fractal geometries in real-world settlement patterns (as attempted in Nguyen et al.^12^) made such an attempt, but was constrained by the largely manual determination of spatial metrics and thus a limited data volume available for analysis.

The improved procedure presented here has great potential to overcome this limitation, but several choices made along the way still need to be reconsidered in order to unleash the combined potential of graph-based network analysis and fractal theory together. This includes in particular:

- *Size classification*: The clustering algorithm used here^23^ required fixing the number of classes in advance, and it was (arbitrarily) set to five classes. In hindsight the data indicated that four classes might have been more appropriate for the Punjab and the Nile Delta, and three classes for China. Other algorithms should be explored that set the break threshold so that the number of finally resulting classes becomes flexible.
- *Connection distance*: The use of fixed buffer sizes is not generalizable and the chosen settings may therefore not be suitable for every region of the world. In an improved version of the approach, we plan to automatically determine the initial search buffers for connecting neighboring settlements of the same size class by using 10 times the average diameter of all settlements in the target class to be connected. In a second analysis run based on this initial network, the distance relationships (rather than the size characteristics) between all objects of the same settlement size class are determined exactly and the distance values obtained are used to optimize the initially generated network (the objects of the same size class remain connected – or will be connected - only if the distance between them is a maximum of e.g. the average distance between the objects of this size class or the average applied with a factor). With such a two-stage, adaptive definition of buffer distances, the resulting final networks should fit reasonably well in all regions of the world and become well comparable. Such could serve as a basis for the assessment of their fit to fractal patterns.
- *Number of connections*: To keep the complexity of the generated network and the required computing manageable even for very large study areas, the number of connections has to be reduced. This will allow to increase the informative value of the connections. Currently, all settlement objects within the search buffer are connected. With the iterative thinning proposed above, their number should decrease considerably. If this is not sufficient, this step shall be streamlined further, such as by using a combination of Voronoi diagrams and Delaunay triangulation or Thiessen polygonization.
- *Graph analysis*: Adding of new and/or optimization of existing features (e.g., integration of minimum spanning tree or routing routines).
- *Fractal analysis*: Once suitabe parameters for generators, distances and angles are extracted automatically from the network analysis, the application of the Fractalopolis model itself can be further automated. The flexibility offered by the model to rotate axes or gradually adapt the generator between subsequent analysis levels, shall then be explored to better fit the analysed settlement pattern at the first stage rather after iterations as in this study.

While our work shows that in principle the automated analysis of settlement patterns is feasible, a re-evaluation under the above-mentioned modified conditions will be targeted in a subsequent study to further generalize the method for extracting hierarchical relationships and assess the fractal features of the settlement pattern. As fractal patterns, by definition, are self-similar across scales, this analysis should be applicable at any spatial scale.

Urbanization is a process that proceeds over time, and can be traced by the analysis of multitemporal satellite images. It remains to be examined if the settings such as size classifications and connection distances optimized for a present stage are applicable also to older pictures of the same study region, or if they have to be optimized for each time point.

At the lowest size class of settlements, we have noted a deviation from fractal hierarchies. It would be worthwhile to substantiate the apparently even distribution of these settlements by spatial metrics, as it represents a fundamentally different pattern. In any case, thinking in terms of patterns, their potential overlays and mutual modulations is a way of moving away from the prevailing city-centered. It will help to understand generic structures of *rurban* settlement patterns, their dynamics, and underlying drivers.

## Electronic supplementary material

Below is the link to the electronic supplementary material.

Supplementary Material 1

## Data Availability

Datasets used in this study are available here: https://download.geoservice.dlr.de/WSF3D/files/. All data generated or analysed are available on reasonable request from corresponding author.
