# Supplementary material for: Towards large-scale analyses of settlement patterns in urbanizing landscapes—findings of first studies for India, Egypt, and China
Source: Sci Rep. 2024 Oct 28;14:25782. doi: 10.1038/s41598-024-74678-w (PMC11519479; doi:10.1038/s41598-024-74678-w)

Towards large-scale analyses of settlement patterns in urbanizing landscapes—findings of first studies for India, Egypt, and China

Thanh Thi Nguyen[a,*], Thomas Esch[b,d], Ellen Hoffmann[a], Julian Zeidler[b], Lorenz Gruber[c], Dennis Kaiser[b], Andreas Buerkert[a]

[a]Organic Plant Production and Agroecosystems Research in the Tropics and Subtropics, Faculty of Organic Agricultural Sciences, University of Kassel, 37213 Witzenhausen, Germany
[b]German Aerospace Center (DLR), German Remote Sensing Data Center, Land Surface Dynamics, Oberpfaffenhofen, 82234 Weßling, Germany
[c]Department of Computer Science, University of Wuerzburg, 97074, Wuerzburg, Germany
[d] Faculty of Geomatics, Computer Science and Mathematics Photogrammetry and Geoinformatics , University of Applied Sciences (HFT) , Schellingstr. 24, 70174, Stuttgart , Germany

[*] corresponding: tropcrops@uni-kassel.de

Annex: Example of automatically generated settlement network (A), a manually defined network in Nguyen et al. (2022) (B), and defined settlement hierarchy view all patches (C) in the Punjab region, India. Map (A) and (C) were generated from QGIS 3.22, map (B) was generated from QGIS 3. 16

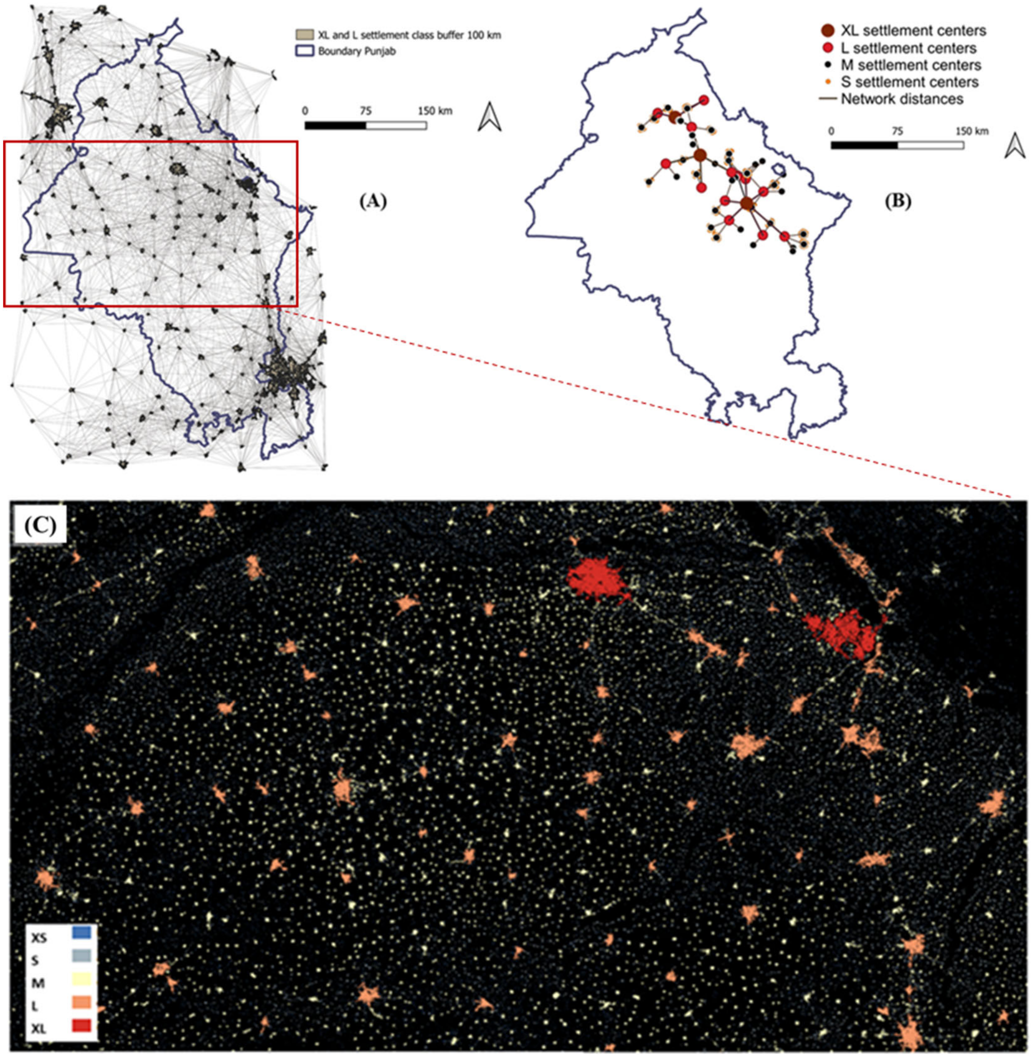

Supplement: Supplementary file 1 — Supplementary Material 1 [file 41598_2024_74678_MOESM1_ESM.pdf]
